# Late Life-Threatening Hemorrhage after Percutaneous Tracheostomy

**DOI:** 10.1155/2011/890380

**Published:** 2011-04-14

**Authors:** Torsten Richter, Birgit Gottschlich, Susanne Sutarski, Rainer Müller, Maximilian Ragaller

**Affiliations:** ^1^Department of Anesthesiology and Intensive Care Medicine, Dresden University of Technology, Carl Gustav Carus University Hospital, Fetscher Street 74, 01307 Dresden, Germany; ^2^Department of Otorhinolaryngology, Bavaria Clinic, An der Wolfsschlucht 1-2, 01731 Kreischa, Germany; ^3^Department of Otorhinolaryngology, Dresden University of Technology, Carl Gustav Carus University Hospital, Fetscher Street 74, 01307 Dresden, Germany

## Abstract

*Purpose.* Formation of a tracheoinnominate artery fistula (TIF) and consecutive hemorrhage is a rare and life-threatening complication with high mortality. Warning symptoms can be absent. The current literature contains only few considerations for misleading signs, especially in cases where the contact between the tissue and the cannula is tight. *Method and Results.* We report two cases of life-threatening hemorrhages that appeared six days and two months after percutaneous dilatational tracheostomy (PDT) in two patients, respectively. In these cases, diagnosis of tracheoinnominate artery fistula (TIF) was difficult. Tracheal ring fracture after PDT and pressure ulceration caused by cannula were implicated in TIF formation. The cannula was overblocked to buy time before surgical closure. Both patients survived without any additional neurological deficiency. *Conclusion.* Massive hemorrhage in patients after tracheostomy is likely due to TIF. Ultrasound scanning before PDT and careful periodical followup of the trachea are required.

## 1. Introduction

Brachiocephalic artery hemorrhage is a life-threatening complication with high mortality [1, 2]. Fortunately, TIF is rare, with a reported incidence between 0.1–1% after surgical tracheostomy and 0.35% after PDT [3]. Delayed hemorrhage caused by TIF following PDT is assumed to be underreported [4]. 

## 2. Case Reports

**Case 1**

**Figure 1:**
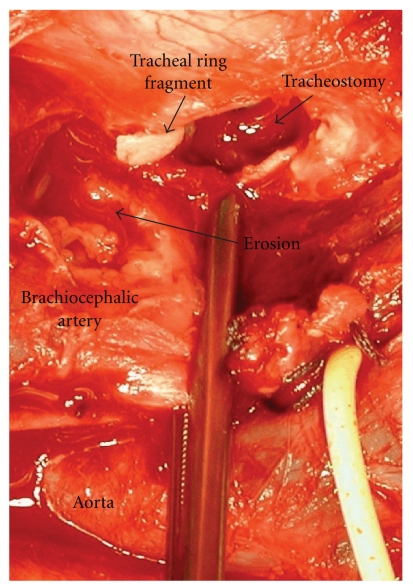
Intraoperative view (Case 1). Please note the tip of the tracheal ring fragment near the dorsal wall lesion of the brachiocephalic artery.

A 32-year-old female was admitted because of intracerebral bleeding due to a high-flow arteriovenous malformation of the posterior inferior cerebellar artery. It was treated by neuroradiological intervention and neurosurgical extirpation. Because of prolonged ventilation, translaryngeal tracheostomy using the Fantoni technique was performed. Excessive oropharyngeal hemorrhage occurred six days later, during a routine bedding procedure. The otorhinolaryngologist could not identify the source of bleeding and placed a nasopharyngeal, oropharyngeal and hypopharyngeal tamponade. Blood loss was reduced, and it stopped completely after about 15 minutes. Angiographic computer tomography could not show the source of bleeding. Two hours later, a second massive hemorrhage occurred from the oropharynx beside the tamponade and tracheal tube. The tube was overblocked extensively. A second attempt to identify the source of bleeding by angiography failed. The patient was transported to the operating room for revision of tracheostomy. An erosive lesion in the dorsal wall of the brachiocephalic artery was found (Figure 1) and closed. PDT was replaced by conventional tracheostomy. The following postoperative period was uneventful.

**Case 2**

**Figure 2:**
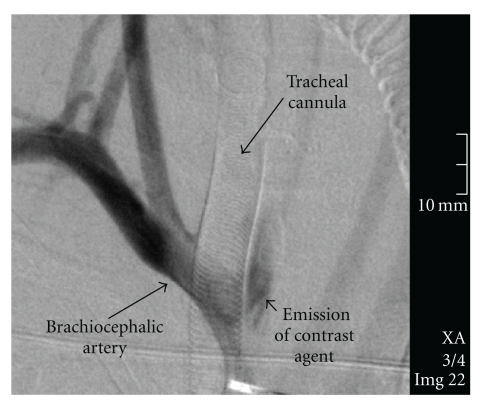
Angiographic scan (Case 2) of a free contrast agent beside the brachiocephalic artery at the level of the left lateral circumference of the tracheal cannula after slight tracheal cuff deflation.

A 19-year-old male, who had suffered severe head and thorax trauma several months ago, was presented to an otorhinolaryngologist. A Griggs PDT was performed two months ago. Around the tracheostoma was a thick scab of old blood. For a diagnostic tracheoscopy, the otorhinolaryngologist removed the tracheal speech cannula. Sudden excessive bleeding occurred from the nose and mouth. A cuffed tracheal cannula was inserted and overinflated immediately to prevent the aspiration of blood by the suspected nasopharyngeal bleeding. A tamponade of the oropharynx, nasopharynx and hypopharynx was given, and volume resuscitation was started. After transportation to the university hospital, a second severe hemorrhage occurred. Angiography showed no source of bleeding. After slight tracheal cuff deflation an escape of the contrast agent was observed from the proximal brachiocephalic artery (Figure 2). Reconstruction of the brachiocephalic trunk was performed immediately, and the tracheostomy was closed. The patient was extubated two weeks after this operation and was then transferred back to the rehabilitation clinic one week later.

## 3. Discussion

Brachiocephalic artery hemorrhage is a life-threatening complication after tracheostomy. Warning symptoms, such as aspiration of blood, bleeding beside the tracheal cannula, or pulsation of the cannula, can be absent. The first signs of bleeding can be misleading and occur as a naso-oropharyngeal hemorrhage, especially in cases where the contact between the tissue and the cannula is tight. Fiberoptic bronchoscopy and angiography have been performed with mixed results [3]. Angiography showed the bleeding source only with slightly reduced cuff pressure in the second case. Therefore, TIF is a likely cause of massive hemorrhage in patients after tracheostomy, until proven otherwise. 

As massive hemorrhage begins, immediate arterial compression by overinflation of the cuff, control of the airway, and subsequent surgical treatment of the injured artery were lifesaving.

In the first case, the translaryngeal tracheostomy produced a tracheal ring fracture. By extraction of the cone cannula during the PDT using the retrograde Fantoni technique [5], a tracheal ring fragment was undetectably pulled towards the artery. Hereby, variation in the position of the brachiocephalic artery with respect to the trachea has to be taken into account [6]. Fracture of tracheal cartilage is described to be responsible for consistently increased cuff pressure [1] and local inflammation [7]. Ongoing mechanical irritation from the tracheal cartilage fragment and transmitted pressure from the tracheal cannula eroded the wall of the trachea and the brachiocephalic artery after only six days. 

Ultrasound examination detects the anatomical relation of major vessels to the trachea [8] and may, therefore, be preventive against TIF. Consequently, ultrasound examination should be established routinely to detect anatomical variations before PDT.

TIF formation was reported after long-term pressure due to a “Tracheosafe” device [2] or a cannula [1, 9, 10]. Pressure ulcerations from local inflammation, radiotherapy, high cuff pressure, mucosal trauma due to inappropriately-sized cannula, and malpositioned cannula tip due to atypical head and neck positions of the patient (as seen in severe neurological disorders) may be responsible for TIF formation even months after tracheostomy. Therefore, adequately sized cannula should be used. If a cuffed cannula is required, the cuff pressure should be as low as possible and variations of cuff position should be aspired.

## 4. Conclusion

TIF is a likely cause of massive hemorrhage in patients after tracheostomy. Overinflation of the cuff was lifesaving in both cases. Ultrasound examination was established at our ICU to detect anatomical variations before PDT.

Due to the life-threatening nature of the hemorrhage, fiberoptic tracheoscopy is required regularly in patients after tracheostomy for early detection of endotracheal injuries, such as tracheal ulceration and pressure necrosis.
